# Carbon reserves in coffee agroforestry in the Peruvian Amazon

**DOI:** 10.3389/fpls.2024.1410418

**Published:** 2024-12-12

**Authors:** Geomar Vallejos-Torres, Nery Gaona-Jimenez, Roger Pichis-García, Luis Ordoñez, Patricia García-Gonzales, Aníbal Quinteros, Andi Lozano, Jorge Saavedra-Ramírez, Juan C. Tuesta-Hidalgo, Keneth Reategui, Wilder Macedo-Córdova, Juan R. Baselly-Villanueva, César Marín

**Affiliations:** ^1^ Escuela Profesional de Agronomía, Universidad Nacional de San Martín, Tarapoto, San Martín, Peru; ^2^ Salud Agroforestal, Instituto de Investigaciones en Salud Agroforestal (IISA), Tarapoto, Peru; ^3^ Escuela Profesional de Ingeniería Ambiental, Universidad César Vallejo, Tarapoto, San Martín, Peru; ^4^ Facultad de Ingeniería y Ciencias, Universidad Nacional Autónoma de Alto Amazonas, UNAAA, Yurimaguas, Peru; ^5^ Facultad de Ingeniería y Ciencias Ambientales, Universidad Nacional Intercultural de la Amazonía, Pucallpa, Peru; ^6^ Facultad de Zootecnica, Universidad Nacional de la Amazonia Peruana, Iquitos, Peru; ^7^ Área de Ciencias Forestales, Instituto Nacional de Innovación Agraria - INIA, Iquitos, Maynas, Peru; ^8^ Centro de Investigación e Innovación para el Cambio Climático (CiiCC), Universidad Santo Tomás, Valdivia, Chile; ^9^ Amsterdam Institute for Life and Environment, Section Ecology & Evolution, Vrije Universiteit Amsterdam, Amsterdam, Netherlands

**Keywords:** agroforestry, carbon stocks, Peruvian Amazon, secondary forests, shade trees

## Abstract

**Introduction:**

Secondary forests and coffee cultivation systems with shade trees might have great potential for carbon sequestration as a means of climate change adaptation and mitigation. This study aimed to measure carbon stocks in coffee plantations under different managements and secondary forest systems in the Peruvian Amazon rainforest (San Martín Region).

**Methods:**

The carbon stock in secondary forest trees was estimated using allometric equations, while carbon stocks in soil, herbaceous biomass, and leaf litter were determined through sampling and laboratory analysis.

**Results:**

The biomass carbon stock in secondary forests was 132.2 t/ha, while in coffee plantations with *Inga* sp. shade trees was 118.2 t/ha. Carbon stocks were 76.5 t/ha in coffee with polyculture farming, while the lowest amount of carbon was found in coffee without shade trees (31.1 t/ha). The carbon sequestered by coffee plants in all agroforestry systems examined had an average of 2.65 t/ha, corresponding to 4.63 % of the total carbon sequestered, being the highest stored in the coffee system with *Inga* sp. shade trees. A higher content of glomalin-related soil proteins (GRSP) was found in coffee without shade trees, with 18.5 mg/g.

**Discussion:**

These results point to *Inga* sp. as a compatible model of shade system for coffee farms. However, broader-scale time-average measurements and carbon dioxide emissions should be assessed in these study systems to have a full understanding of their climate impacts.

## 1 Introduction

Despite multiple efforts and studies, there is still no balanced consensus on the impact of agronomic intensification on shade trees (Haggar et al., 2021). This agroforestry tool could be highly threatened due to global climate change, challenging farmers to maintain agricultural production levels in the future (Gomes et al., 2020). Nevertheless, agroforestry has great climate change mitigation potential, particularly since carbon storage and biodiversity conservation payments are of special interest to coffee farmers (Koutouleas et al., 2024). In particular, the importance of coffee agroforestry systems to preserve biodiversity and ecosystem services has been recognized in different parts of the world (Hundera et al., 2013; Solis et al., 2020). Since coffee shrubs are perennial crops, coffee-based agroforestry practices are believed to have higher biomass carbon sequestration, biodiversity, and ecosystem function than other agroforestry practices (Buechley et al., 2015; Hylander et al., 2013; Tesfay et al., 2022).

There is limited knowledge about the effects of arbuscular mycorrhizal fungi (AMF) on carbon sequestration in agroforestry systems across different agroecological settings (Tschora and Cherubini, 2020). Such a role of AMF can be mediated or explained by their production of glomalin. Nautiyal et al. (2019) highlighted the importance of glomalin in maintaining soil aggregation and its positive correlation with soil organic carbon (SOC), which not only increases total carbon stock but also binds to soil organic matter (SOM), preventing soil erosion and further improving its aggregation. At the same time, AMF contribute to a greater extent to regulating glomalin and glomalin-related soil protein (GRSP) contents, which are important indicators of SOC stock (Li et al., 2020; Santos et al., 2022), varying according to the type of agroforestry system (Santos et al., 2022). Thus, Cai et al. (2023) found that GRSP content significantly promoted SOC sequestration. Therefore, GRSP is an important component of the soil carbon pool, as it improves the structure of soil aggregates. Likewise, GRSP produced by AMF is a carbon reserve that influences the formation and stabilization of aggregates and contributes to soil carbon sequestration (Nautiyal et al., 2019). Therefore, there is an urgent need to study the potential impact of glomalin secreted by AMF in contributing to carbon sequestration in forests of the Peruvian Amazon.

Secondary forests are important carbon sinks, absorbing CO_2_ from the atmosphere through photosynthesis and photosynthate storage in their aboveground living biomass. Therefore, healthy secondary forests are well-adapted and constitute efficient carbon sinks whose conservation is vital to mitigate and adapt to climate change and to support biodiversity (Griscom et al., 2020; Aragón et al., 2021). They also have higher CO_2_ sequestration rates than costly and poorly adapted afforestation and reforestation initiatives (Chazdon and Guariguata, 2016).

Despite the potential of secondary forests and coffee agroecosystems for carbon sequestration, they are almost completely ignored in land use management in Latin America. In Peru, these Amazonian ecosystems could help to reach the country’s compromises on climate change adaptation and mitigation. Peru committed to restoring 3.2 million hectares of forests by 2030, of which 2 million hectares will be restored through commercial plantations—including naturalized and non-native species. However, in these commitments, secondary forests were not explicitly included. Such commitments were made aiming at reducing emissions by 30% (Aragón et al., 2021).

In the Peruvian Amazon, there is little research on above- and belowground carbon variations in agroforestry systems with coffee and shade trees. Overall, the Amazon rainforest has barely been investigated regarding its mycorrhizal biodiversity and functioning (Marín et al., 2022). Therefore, this exploratory study aimed to quantify carbon stocks in coffee plantations under different agricultural managements and secondary forest systems in the Peruvian Amazon rainforest. However, these measurements correspond to a single measurement in time and should be followed through several years and also include carbon dioxide emissions.

## 2 Materials and methods

### 2.1 Study area

**Figure 1:**
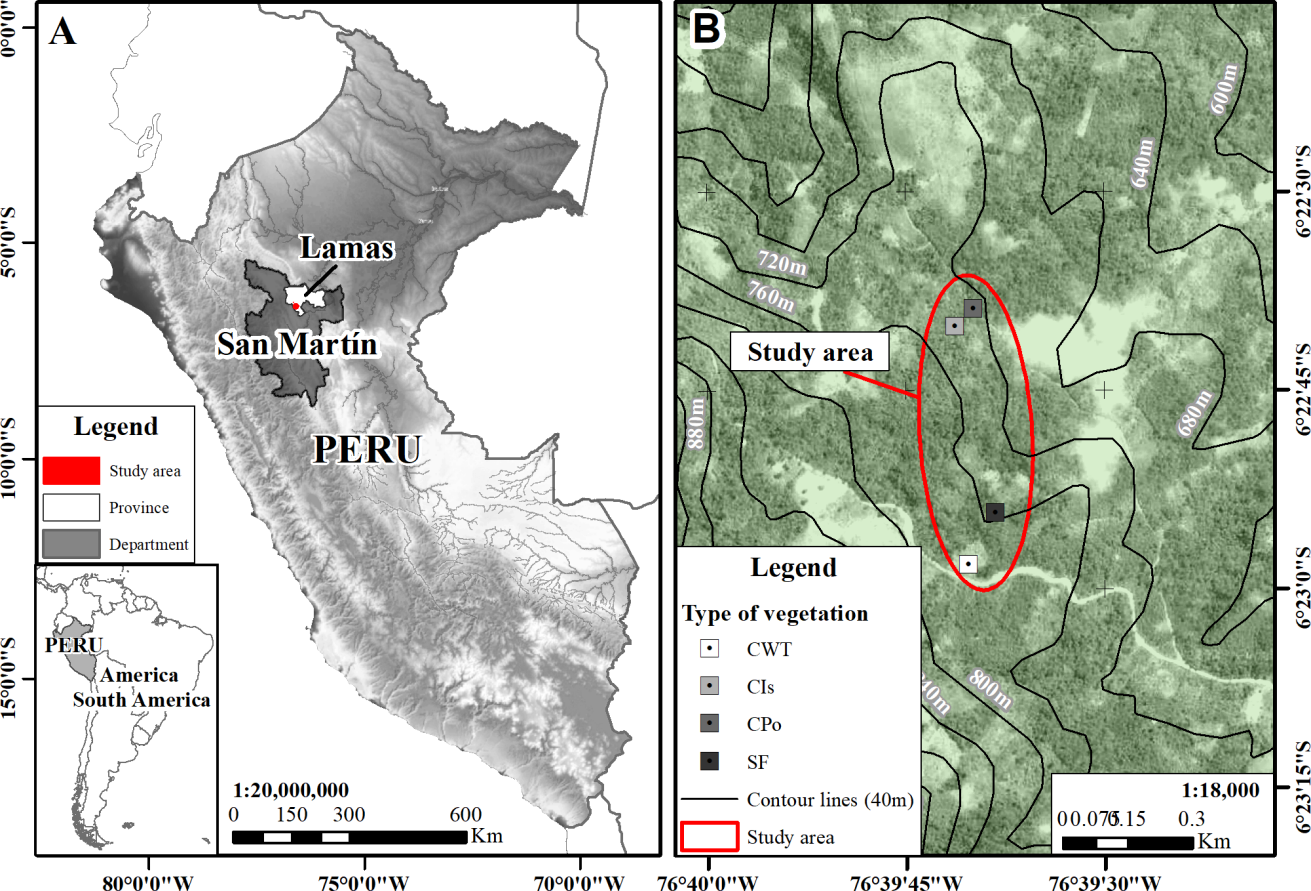
Study site location. **(A)** San Martín region in the Peruvian Amazon. **(B)** Plots of studied coffee agroforestry and secondary forest.

The study was carried out in the annexes of the Tabalosos District in the province of Lamas, San Martín region in the Peruvian Amazon between September 2022 and January 2023 (**Figure 1**). The province is located at an altitude range between 310 and 814 m.a.s.l. The average annual rainfall and temperature in this district are 1,013 mm and 32°C, respectively, with August and December being the summer months and March to April being the winter months, very similar to the majority of districts in the region.

### 2.2 Sampling design

Four vegetation cover types were identified in three zones with coffee and one zone of secondary forest (**Table 1**). These were as follows: 1) coffee without shade trees (monoculture), that is, coffee plantations with a higher planting density than the other systems (2,000 coffee plants per hectare), made up of the Caturra, Pache, and Catimore varieties (all growing together); 2) coffee with *Inga* sp. shade trees, with a density of 1,500 plants per hectare of the Caturra, Catimore, and Pache varieties (all growing together); 3) coffee with polyculture farming, with a density of 1,450 plants per hectare of the Caturra and Pache varieties, composed of a diversity of agroforestry crops (specified in **Table 1**); and 4) a secondary forest with a diversity of trees, which were more than 15 years old (**Table 1**). Organic coffee farms between 9 and 13 years old (age of coffee trees) were considered, with similar agronomic management between them.

**Table 1:** Forest composition of the types of coffee and secondary forest agroforestry systems.

Type of vegetation cover	Code	Altitude (m.a.s.l.)	Plant species	Basal area (m^2^/ha)	pH	N (%)	P (mg kg^−1^)
Coffee without shade trees	CWT	772	Coffee (*Coffea arabica*) (20 plants per subplot)	7.52	5.42	0.13	1.32
Coffee with *Inga* sp. shade trees	CIs	783	Coffee (*C. arabica*) (15 plants per subplot) and guaba (*Inga* sp.) (10 plants per subplot)	17.03	5.72	0.18	1.32
Coffee with polyculture farming	CPo	704	Coffee (*C. arabica*) (14 plants per subplot) and 15 plants per subplot of cedar (*Cedrela odorata*), avocado (*Persea americana*), and cocoa (*Theobroma cacao*)	2.93	6.75	0.24	2.00
Secondary forest	SF	769	Shimbillo (*Inga* spp.), atadijo (*Trema micrantha*), Shaina (*Colubrina glandulosa*), cetico (*Cecropia sciadophylla*), and zapote (*Manilkara zapota*) (12 plants per subplot)	8.33	6.35	0.18	3.22

Area of each subplot = 100 m^2^.

Following Grünzweig et al. (2003) and Roxburgh et al. (2006) regarding plot size, the biomass and carbon of the trees were evaluated in four plots (one plot per cover type) with an area of 30 m × 30 m with slopes between 0° and 40°. Four subplots of 4 m × 25 m (100 m^2^) were established in each plot to facilitate the evaluation of trees and coffee shrubs, for a total of 16 subplots. The height and diameter of the woody components were measured using a Suunto clinometer and tape measure, respectively. **Table 1** shows the density of the different plants per treatment, per 100 m^2^ subplot. The diameter of coffee plants was measured at 15 cm above the ground (d15) and for trees at 1.3 m above the ground [diameter at breast height (DBH)]. In the same subplots, herbaceous material and litter were measured in a 0.5 × 0.5 m grid according to Siarudin et al. (2021). After removing litter and organic debris, soil was sampled between 0 cm and 15 cm of soil depth, making soil pits in each management system and considering four subplots per system (for a total of 16 soil samples).

### 2.3 Estimation of aboveground carbon

Allometric equations were used to estimate aboveground biomass based on DBH and d15 (**Supplementary Table 1**). Specifically, two allometric equations were used for tropical rainforest species (**Supplementary Table 1**), as one (Nascimento and Laurance, 2002) applies to trees with a DBH of between 2 cm and 5 cm, while the other (Chave et al., 2014) applies to trees with a DBH higher than 5 cm. The wood density value for each identified species was obtained from the “Global Wood Density Database” (Chave et al., 2009). Root biomass was estimated using the regression equations developed by Cairns et al. (1997) (**Supplementary Table 1**). The aboveground and root biomass values obtained for each tree within the same plot were summed to calculate the total tree biomass of the plot, and the result was extrapolated to obtain the biomass stock of 1 ha. Herbaceous biomass was obtained by determining the wet weight of a sample of approximately 500 g; the samples were then taken to the laboratory and dried at 70°C for 48 h to determine the dry weight. With this information, the moisture content and dead biomass were determined using the following equations:


$$\text{Moisture}\;\text{content}=\frac{Wet\;weight\;of\;the\;sample-Dry\;weight\;of\;the\;sample}{Wet\;weight\;of\;the\;sample}$$

$$Dead\;biomass=\sum_{i=1}^{n}\left(Total\;wet\;weight-\left(Total\;wet\;weight\;x\;Moisture\;content\right)\right)$$

Leaf litter biomass was determined in the same way as dead wood biomass. It was assumed that the carbon present in the biomass could be 50% (Intergovernmental Panel on Climate Change - IPCC, 2003). The C content obtained in each sampled component was extrapolated to estimate C stocks per hectare.

### 2.4 Estimation of soil organic carbon

The cylinder method of 5.5-cm diameter and 5-cm height proposed by Blake and Hartge (1986) was used to calculate the bulk/apparent density (AD) of the soil in g/cm^3^, determined by the following formula:


(1) AD:Wd/V

where AD is the apparent density (g/cm^3^), Wd is the weight of the oven-dried soil sample (g), and V is the volume of the sampled soil (cm^3^). Therefore, SOC was determined using the method developed by Walkley and Black (1934) in the laboratory, using the following formula:


(2) SOC (tC/ha)=OC×Pf×DA

where OC is soil organic carbon content (%), Pf is soil sampling depth (cm), and AD is the apparent density (g/cm^3^).

Thus, we determined the weight of sequestered carbon dioxide in the tree by multiplying the tree’s carbon weight by 3.67 (Paniagua-Ramirez et al., 2021).

### 2.5 Estimation of arbuscular mycorrhizal abundance and glomalin content in soils

The quantification of AMF spores was performed by wet sieving and decanting, as proposed by Gerdemann and Nicolson (1963), with some modifications. Likewise, in order to determine GRSP content, soil samples were taken following the methodology of Solis et al. (2022). The total GRSP fraction was extracted according to Wright and Upadhyaya (1998) and quantified by the method of Bradford (1976).

### 2.6 Statistical analyses

The statistical analyses were performed through R Studio (R Core Team, 2023). To analyze the effect of the vegetation cover factor on carbon content, AMF spores, and GRSP content, non-parametric statistics were used to evaluate carbon reserves. The Mann–Whitney test was used to compare two medians with a probability of error of 5%. A principal component analysis (PCA) biplot was performed to evaluate the correlations between variables. The variables were standardized, and the *fviz_pca_biplot* function of the Factoextra package of R (Kassambara and Mundt, 2022) was used for the PCA. A correlogram was also performed to test these correlations, using Pearson’s correlation coefficient (p < 0.05), using the *ggpairs* function of the GGally package in R (Schloerke et al., 2022).

## 3 Results

### 3.1 Carbon storage capacity in coffee and secondary forest

**Figure 2:**
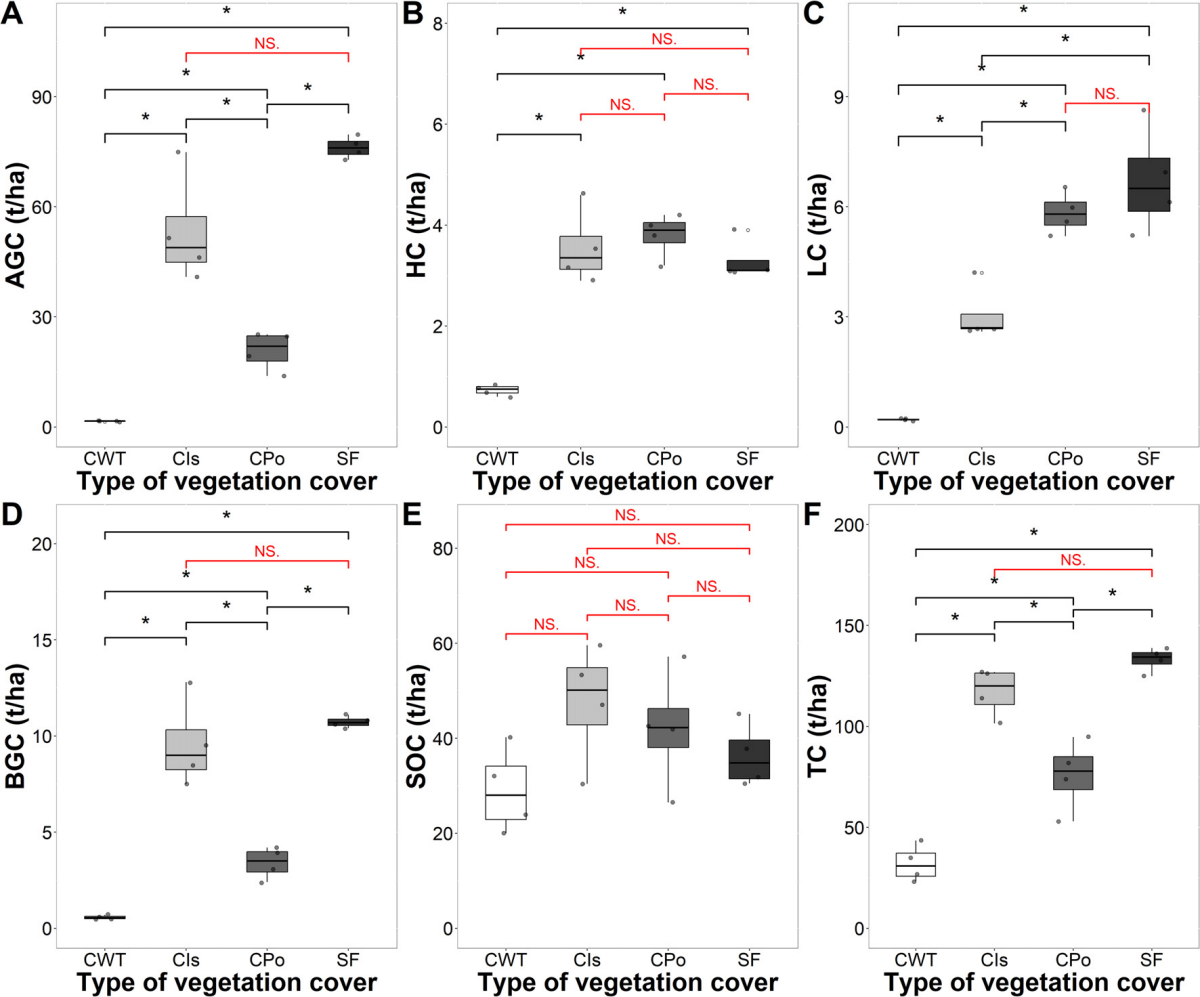
Carbon storage of coffee agroforestry and secondary forest systems by carbon component. **(A)** Carbon storage in the aboveground component (AGC). **(B)** Carbon storage in the herbaceous component (HC). **(C)** Carbon storage in leaf litter (LC). **(D)** Carbon storage in the belowground (BGC). **(E)** Carbon storage in soil organic carbon (SOC). **(F)** Total carbon storage. CWT, coffee without shade trees; Cis, coffee with *Inga* sp. shade trees; Cpo, coffee with polyculture farming; SF, secondary forest. Mann–Whitney test: * at p < 0.05 and NS. (non-significant) at p > 0.05.

Carbon stocks varied according to the type of vegetation cover (**Figure 2**). The overall mean storage of carbon stocks in the different types of vegetation cover was 89.5 t C/ha. The total carbon storage capacity (t C/ha) in the different cover types decreased in the following order: SOC (38.74 C/ha) > aboveground carbon (AGC; 37.96 C/ha) > belowground carbon (BGC; 6.06 C/ha) > litter carbon (LC; 3.93 C/ha) > herbaceous carbon (HC; 2.84 C/ha).

The total carbon stock in the different vegetation cover types ranged from 32.1 t C/ha in coffee without shade trees to 133.08 t C/ha in the secondary forest (**Figure 2F**). The highest carbon content was found in the secondary forest and coffee with *Inga* sp. shade trees with a total of 133.08 and 117.13 t/ha, respectively, being statistically higher than those of the other covers (p < 0.05).

Aboveground carbon stocks varied from 1.58 t C/ha in coffee without shade trees to 76.13 t/ha in the secondary forest (**Figure 2**). Vegetation covers significantly influenced carbon stocks in the different components with the exception of SOC (p < 0.05) (**Figure 2**). The secondary forest and the coffee system with *Inga* sp. shade trees had the highest AGC (76.13 and 53.38 t C/ha) and BGC (10.7 t C/ha) carbon stocks (**Figures 2A, D**). Likewise, coffee without shade trees had the lowest HC and LC at 0.73 and 0.20 t C/ha, respectively (**Figures 2B, C**). With respect to SOC, we found values ranging from 29.05 t C/ha in coffee without shade trees to 45.7 t C/ha in coffee with *Inga* sp. shade trees (**Figure 2E**).

**Figure 3:**
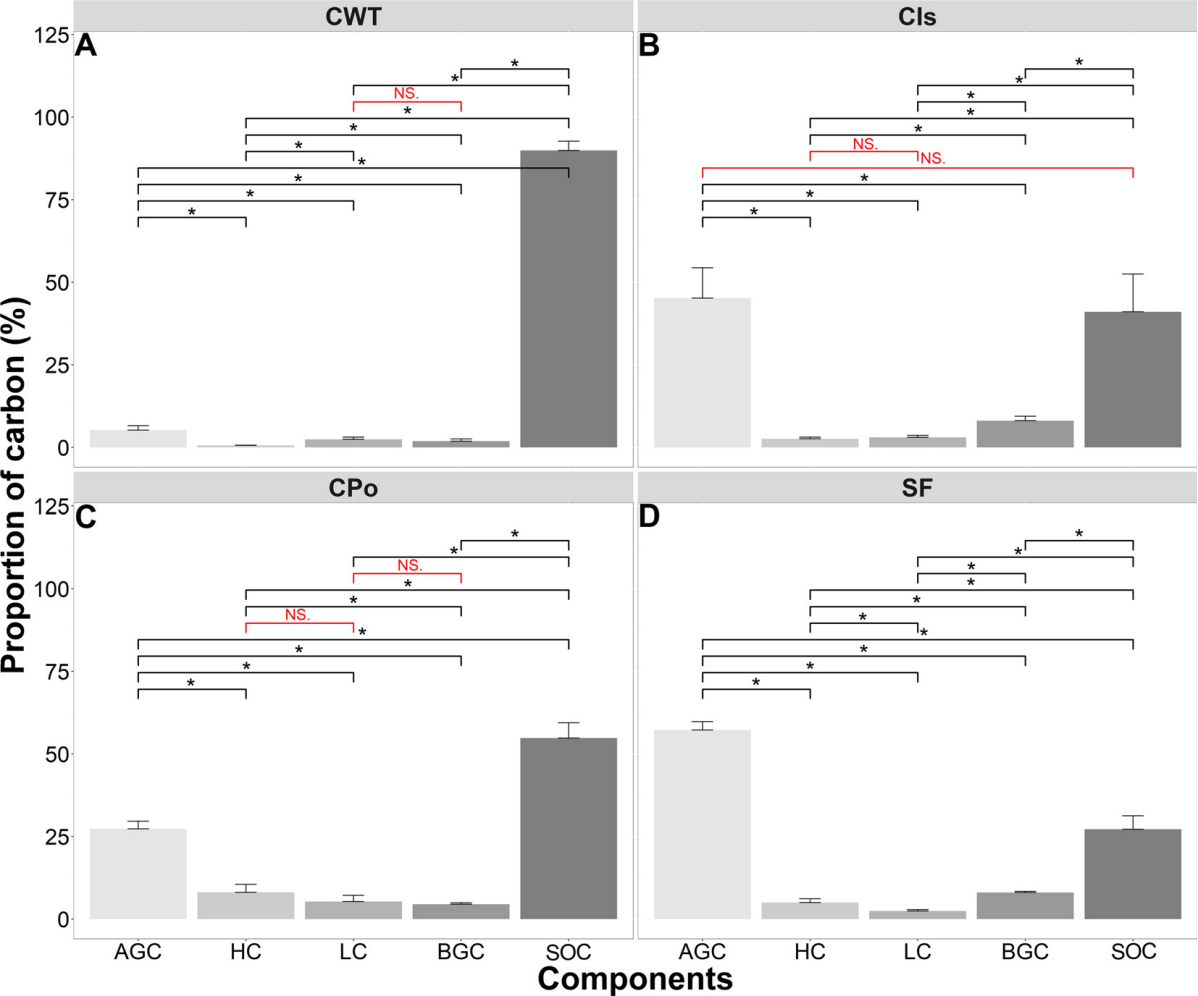
The proportion of carbon components stored in the different types of vegetation cover. **(A)** Proportion of carbon in coffee without shade trees (CWT). **(B)** Proportion of carbon in Coffee with *Inga* sp. shade trees (CIs). **(C)** Proportion of carbon in coffee with polyculture farming (CPo). **(D)** Proportion of carbon in secondary forest (SF). AGC, aboveground carbon; HC, herbaceous carbon; LC, leaf litter carbon; BGC, belowground carbon; SOC, soil organic carbon. Mann–Whitney test: * at p < 0.05 and NS. (non-significant) at p > 0.05.

The proportions of carbon stored in the different vegetation and soil compartments were affected by the different vegetation cover types (p < 0.05). SOC presented the highest proportion of carbon in the three coffee covers (CWT, coffee without shade trees; Cis, coffee with *Inga* sp. shade trees; Cpo, coffee with polyculture farming), while with respect to the secondary forest (SF), the highest proportion was obtained in AGC (**Figure 3**). The SF sequestered 57.27% of carbon as AGC, 8.06% as BGC, 27.19% as SOC, and the rest as LC and HC (**Figure 3D**). The coffee with *Inga* sp. shade trees sequestered 45.17% of carbon as AGC, 41.08% as SOC, and 13.75% as LC, HC, and BGC (**Figure 3B**). Coffee with polyculture farming sequestered 54.81% of carbon as SOC, 27.30% as AGC, 5.32% as LC, and 12.57% as HC and BGC (**Figure 3C**), whereas the coffee without shade trees sequestered 89.90% of carbon as SOC, followed by AGC, HC, LC, and BGC with 5.22%, 0.58%, 2.42%, and 1.88%, respectively (**Figure 3A**).

### 3.2 Glomalin and spore content of arbuscular mycorrhizal fungi

**Figure 4:**
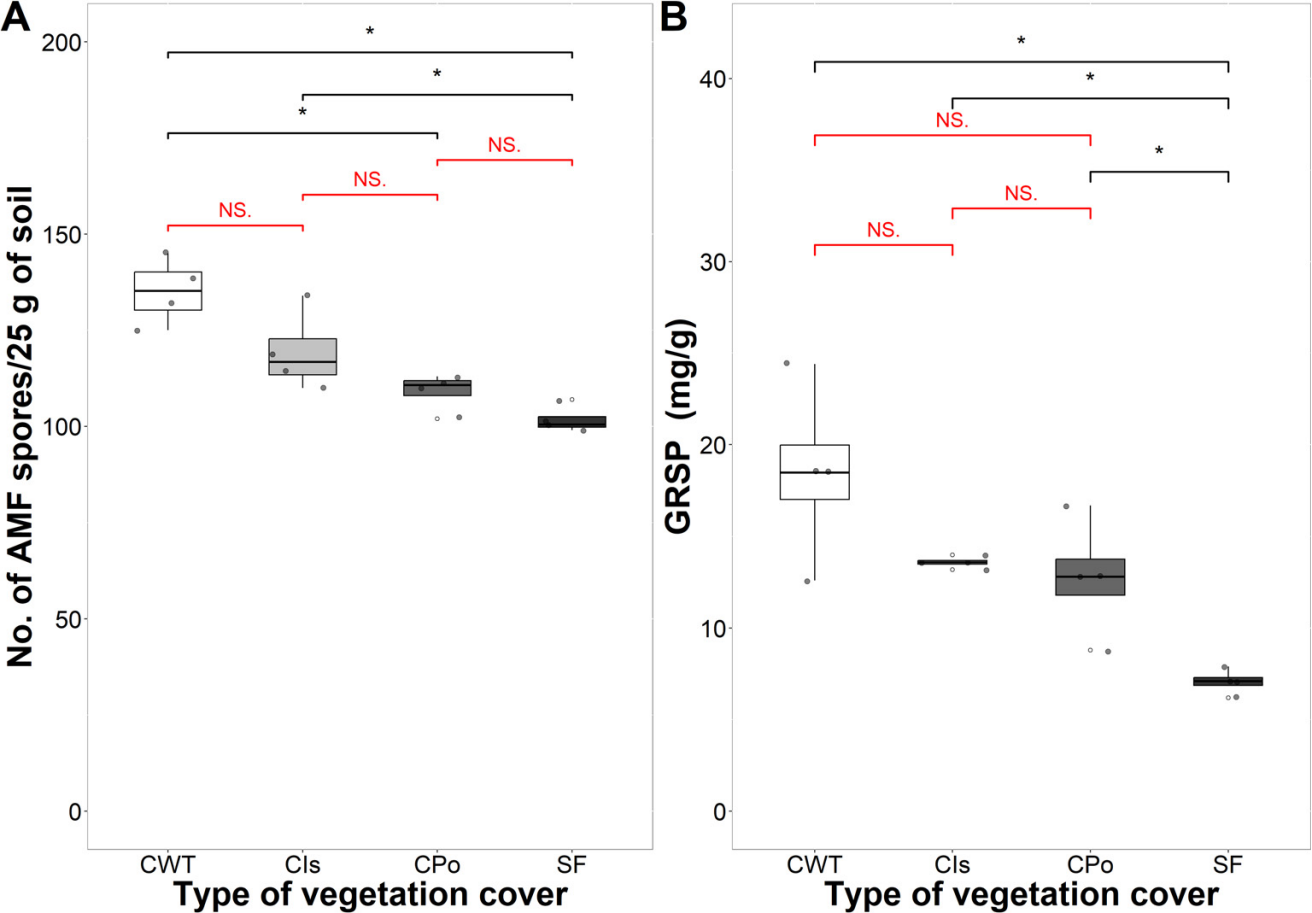
Glomalin in different types of vegetation cover. **(A)** Number of arbuscular mycorrhizal fungal (AMF) spores per 25 g of soil. **(B)** Glomalin-related soil protein (GRSP) (mg/g). CWT, coffee without shade trees; CIs, coffee with *Inga* sp. shade trees; CPo, coffee with polyculture farming; SF, secondary forest. Mann–Whitney test: * at p < 0.05 and NS. (non-significant) at p > 0.05.

The lowest number of AMF spores was found in coffee with polyculture and secondary forest with an average of 110 and 102 spores per 25 g of soil, respectively, showing significant differences compared to coffee without trees (**Figure 4A**). The highest GRSP content was found in coffee without shade trees with an average of 18.50 mg/g, and the lowest content was found in the secondary forest with 7.08 mg/g on average, showing significant differences for both covers (p < 0.05) (**Figure 4B**). In contrast, coffee with *Inga* sp. shade trees and coffee with polyculture farming did not show significant differences in GRSP content among them (**Figure 4B**).

The variation of carbon stocks, number of AMF spores, and GRSP content in the different vegetation covers was explained by 75.8% in the first two principal components of a PCA (**Supplementary Figure 1**). Coffee without shade trees was characterized by the highest values of GRSP and number of AMF spores, variables that were positively correlated (**Supplementary Figures 1**, **2**). Likewise, the secondary forest was characterized by the highest values for AGC, BGC, and LC, variables that showed positive correlations. That is, carbon in aboveground biomass increases as underground biomass and litter biomass increase in the secondary forest cover.

GRSP ranged from 6.22 to 24.36 mg/g, and the number of AMF spores was between 99 and 145 spores per 25 g of soil, variables that presented a significant and positive correlation with an r^2^ value of 0.805. GRSP and the number of AMF spores presented significant and negative correlations with all carbon pools except SOC (**Supplementary Figure 2**). The number of AMF spores and glomalin concentration were influenced by the amounts of carbon stored, mainly by that sequestered in LC (r^2^ of −0.748 and −0.824 for GRSP and the number of AMF spores, respectively).

### 3.3 Carbon sequestration

**Figure 5:**
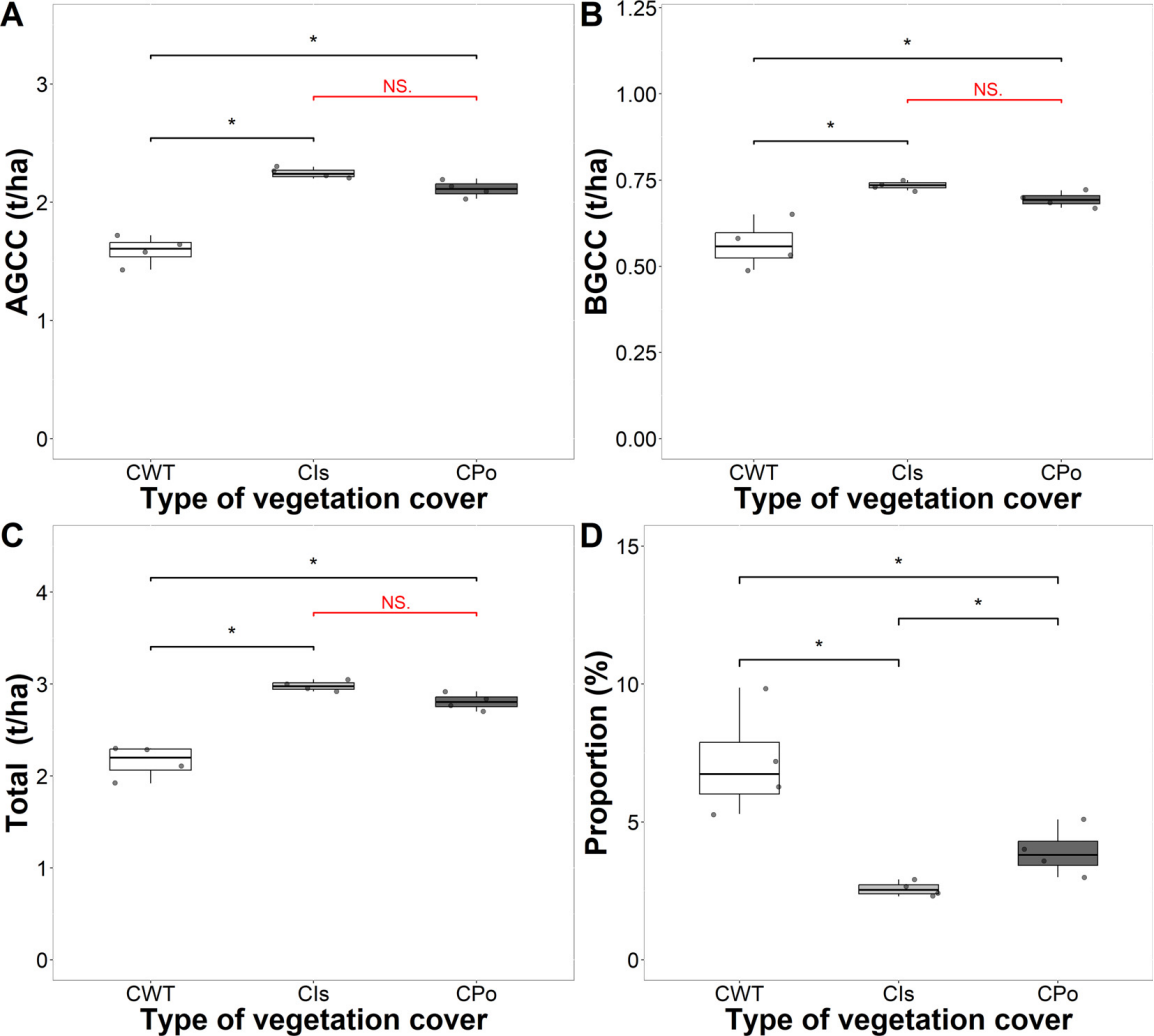
Amount and proportion of carbon stored in coffee plants in the coffee agroforestry systems by carbon component. **(A)** Carbon stored in aboveground coffee (AGCC). **(B)** Carbon stored in belowground coffee (BGCC). **(C)** Total carbon stored in coffee. **(D)** Proportion of carbon stored in coffee plants. CWT, coffee without shade trees; CIs, coffee with *Inga* sp. shade trees; CPo, coffee with polyculture farming. Mann–Whitney test: * at p < 0.05 and NS. (non-significant) at p > 0.05.

Coffee plants also contributed to the total carbon stock in the different types of vegetation cover (**Figure 5**). The coffee plants growing with *Inga* sp. and polyculture presented greater carbon aboveground, belowground, and total carbon compared to coffee without trees (p < 0.05) (**Figures 5A–C**). The mean values of carbon sequestered by coffee plants with *Inga* sp. shade trees and polycultures were significantly different from those of coffee without shade trees but did not differ significantly from each other. The carbon sequestered by coffee plants was 2.98, 2.81, and 2.16 t C/ha in coffee with *Inga* sp. shade trees, coffee with polyculture farming, and coffee without shade trees, respectively (**Table 2**). Coffee plants contributed approximately 7.16%, 3.93%, and 2.57% of the carbon sequestered in coffee without shade trees, coffee with polyculture farming, and coffee with *Inga* sp. shade trees, respectively (**Figure 5D**). The overall average carbon sequestered by coffee plants in the different types of vegetation cover was 2.65 t C/ha (average of 4.63% of total sequestered carbon).

**Table 2:** Total carbon stock and carbon dioxide (CO_2_) equivalent sink in different coffee agroforestry systems and coffee plants.

	Total carbon stock (t/ha)		Equivalent CO_2_ sink (t/ha)	
Type of vegetation cover	In the whole coffee agroforestry	In the coffee plants	In the whole coffee agroforestry	In the coffee plants
CWT	2.16	2.16	7.93	7.93
CIs	117.13	2.98	429.87	10.94
CPo	75.83	2,81	278.3	10.31
Total	195.12	5.14	716.1	29.18

CWT, coffee without shade trees; CIs, coffee with *Inga* sp. shade trees; CPo, coffee with polyculture farming.

In the study area, coffee agroforestry systems sequestered a large amount of carbon in vegetation, including coffee plants and soils proportional to the area of each stratum; approximately 117.13, 75.83, and 2.16 t C/ha were stored in coffee cover with *Inga* trees, polyculture, and coffee without shade trees, respectively (**Table 2**). This shows that a total of 195.12 t C/ha was stored in all coffee agroforestry systems in the study area. Particularly, coffee plants added a total of 7.93 t C/ha in the coffee agroforestry systems (**Table 2**). All coffee agroforestry systems in the current study area trapped 716.1 t of CO_2_ from the atmosphere, while coffee plants captured 29.18 t of CO_2_ from the atmosphere and stored it as carbon in the agroforestry systems.

## 4 Discussion

The higher carbon content present in the secondary forest was due to higher aboveground biomass and population density of the tree species present, as also found by Birhane et al. (2020), who observed that the least disturbed plant community, which had a higher density of trees and shrubs, had the highest SOC stocks compared to more disturbed communities. Secondary forests are of great importance due to the conservation and greater diversity of timber and non-timber trees present, storing more carbon than coffee crops. Soils that form under forests tend to accumulate high levels of organic carbon near the surface and have lower levels of carbon in the subsoil (Girmay et al., 2008). Cover types affected the proportion of carbon stocks stored in different compartments. The results indicate that the highest carbon content was stored as AGC followed by SOC.

Interestingly, regarding the different proportions of carbon components, the secondary forest and coffee with *Inga* sp. shade threes behaved similarly. These are surprising results since in our study, coffee systems were less diverse (coffee, 15 plants; *Inga* sp., 10 plants) than secondary forests (12 trees of different species; **Table 1**). However, coffee plots had higher total tree density and double the basal area (**Table 1**). This may be explained because both are ecosystems in a restoration phase with semi-woody species such as *Inga* sp. and forest species from the secondary forest. Also, both cover types had the same nitrogen content (0.18%) in their soils, which contributes to or may explain their similar SOC contents (and carbon stock proportions) due to the interconnection between N and C (Mehnaz et al., 2018; Jing et al., 2021). However, this good performance of the secondary forest (i.e., the other three subplots had approximately half of the aboveground carbon than the secondary forest) is most probably because each vegetation cover had just one 100-m^2^ subplot.

The highest number of AMF spores was found in coffee without shade systems, where the soils presented the lowest values of pH, total N, and available P with averages of 5.42, 0.13%, and 1.32 mg kg^−1^, respectively (**Table 1**). The larger population of coffee plants present in the coffee system without shade trees probably favors a symbiotic association between coffee plants and AMF, promoting favorable living habitats for the survival and massive multiplication of AMF spores (Vallejos-Torres et al., 2022). In contrast to previous studies (Wang et al., 2017; Singh et al., 2022; Fall et al., 2022), we did not find significant correlations between the number of AMF spores/GRSP and SOC, but we did find such significant (negative) correlations with BGC. In response to mycorrhization, coffee plants show a clear reorganization of the main metabolic pathways, which involve nutrient acquisition, carbon fixation, and primary and secondary metabolism, particularly under low phosphorus conditions (Chialva et al., 2023). This makes coffee a highly mycotrophic plant (Hernández-Acosta et al., 2021).

Our results on higher number of AMF spores on coffee without shade are analogous to those by Polo-Marcial et al. (2023), who found that the abundance and richness of AMF, especially glomerospores, were higher in agroforestry systems than in secondary forests. The presence of AMF plays an important role in the accumulation of GRSP, a critical component of the hyphal cell wall (Wang et al., 2023). Therefore, mycorrhizal hyphae prominently promote enhanced accumulation and preservation of organic carbon in aggregates and soil C pool (Wang et al., 2023). This may have contributed to soil carbon accumulation in coffee without shade trees with 28.0 t/ha.

The carbon stock found in coffee with *Inga* sp. shade trees (125.3 t/ha) was lower than the average carbon stocks reported for agroforestry systems with coffee by Niguse et al. (2022) in Ethiopia (287.1 t C/ha), by Schmitt-Harsh et al. (2012) in Guatemala (259 t C/ha), and by Soto-Pinto et al. (2010) in Mexico (213.80 t C/ha). Similarly, the average SOC content found in this study for the coffee system with *Inga* sp. shade trees (52.8 t/ha) was lower than that reported by Niguse et al. (2022) (91.5 t C/ha) and lower than that reported by Solis et al. (2020) (123.5 t C/ha), both with different shade trees than in our study. Differences in carbon stocks observed in different parts of the world could be attributed to a variety of factors, including coffee variety, management practices, plantation ages, and site factors such as climate and soil conditions. Overall, our findings show that coffee agroforestry systems could sequester a substantial amount of carbon by trapping CO_2_ from the atmosphere, which may help mitigate the effects of climate change (Niguse et al., 2022).

We also investigated the relationships between glomalin and SOC stocks. Glomalin is a soil component potentially produced by AMF; in turn, glomalin is a mixture of soil organic materials that are not unique to AMF (Irving et al., 2021; Holátko et al., 2021). Despite disagreements on the nature of “glomalin” (Holátko et al., 2021), it has been consistently associated with a long list of plant and soil health benefits, including soil aggregation and aggregate stability, soil carbon storage, and improved plant growth under abiotic stress (Plaza et al., 2013; Six and Paustian, 2014; (Nautiyal et al., 2019; Irving et al., 2021). Meanwhile, AMF has a positive impact on soil by producing organic acids and glomalin, which enhance carbon sequestration and stabilize soil macroaggregation (Fall et al., 2022). Likewise, Hawkins et al. (2023) confirmed the important contribution that mycorrhizal hyphae make to global soil carbon dynamics, as they fix—at least temporarily—an equivalent to ~36% of current annual CO_2_ emissions from fossil fuels.

### 4.1 Limitations

Carbon stocks varied according to the type of vegetation cover, with the secondary forest cover storing the highest carbon content, followed by the coffee system with *Inga* sp. shade trees, coffee with polyculture farming, and, to a lesser extent, the coffee system without shade trees. Secondary forests growing on previously cleared land could be a low-cost climate change mitigation strategy due to their potential to sequester CO_2_ (Elias et al., 2022). However, our results may differ if measurements are conducted over several years or if alterations to secondary forests (such as cutting them down) increase their CO_2_ emissions abruptly. Also, we had just one plot (and four subplots) per study system, so an increase in replications or plot size could change the results and their interpretation, particularly since many studies use a 20 × 50 m plot size (Somarriba et al., 2013; Goodall et al., 2015). Thus, these results should be interpreted as they are: a single point in time measurements of carbon stocks is still comparable, as they were conducted at the same time and following the same methodology.

## 5 Conclusions

Coffee plants associated with agroforestry systems sequester substantial amounts of carbon and have AMF that generate glomalin-related soil protein since coffee is a highly mycotrophic plant. That is, coffee benefits from the mycorrhizal association, contributing greatly to the accumulation of carbon in the soil. The carbon sequestration potential of coffee plants with *Inga* sp. shade trees is influenced by the compatibility of the trees present in the management system. Similarly, secondary forest plantations had very high carbon sequestration potential. Thus, our results should not be interpreted as a simplistic recommendation to prioritize coffee plantations with *Inga* sp. over secondary forests. Previous malpractices based solely on carbon capture have had negative effects on local biodiversity, including flora, fauna, and funga. In order to fully understand carbon stocks and their variability in these endangered ecosystems, we suggest follow-up studies on carbon stocks and emissions across different land uses in the Peruvian Amazon rainforest, which can lead to proper policy recommendations.

## Data Availability

The data sets generated during and analysed during the current study are available in the figshare repository: DOI: 10.6084/m9.figshare.27897243.
