# Supplementary material for: Carbon reserves in coffee agroforestry in the Peruvian Amazon
Source: Front Plant Sci. 2024 Dec 12;15:1410418. doi: 10.3389/fpls.2024.1410418 (PMC11670197; doi:10.3389/fpls.2024.1410418)
Supplement: Supplementary file 1 [file DataSheet1.pdf]

Supplementary Material

1 Supplementary Data

Does not apply.

2 Supplementary Figures and Tables

2.1 Supplementary Figures

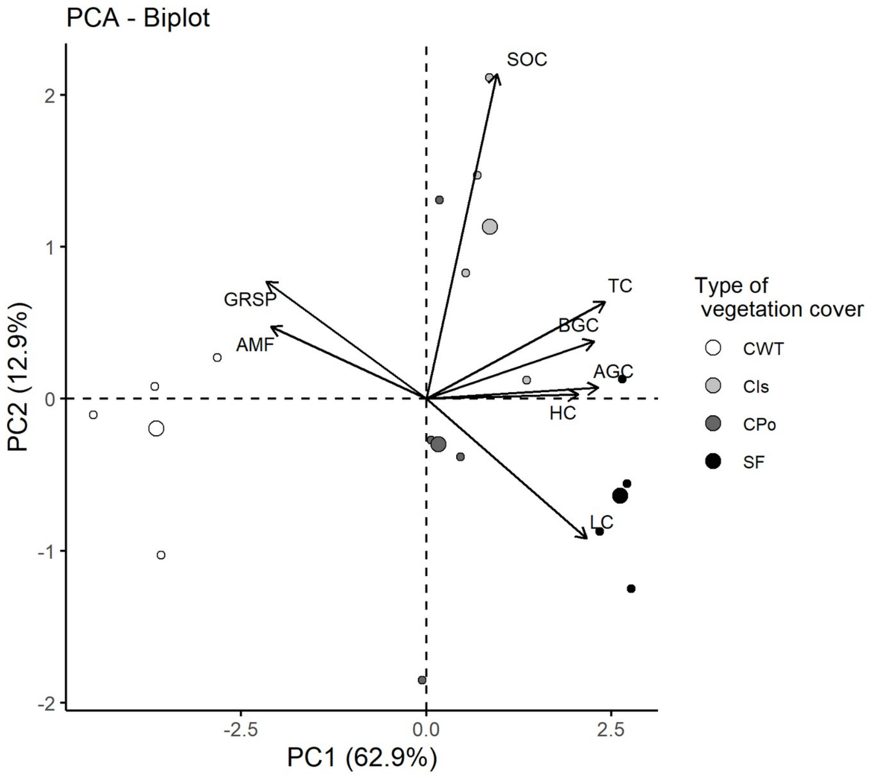

Supplementary Figure 1. Principal Component Analysis of glomalin and mycorrhizae with carbon components. CWT: Coffee without shade trees; CIs: Coffee with *Inga* sp. shade trees; CPo: Coffee with polyculture farming; SF: secondary forest. AGC: aboveground carbon; HC: herbaceous carbon; LC: leaf litter carbon; BGC: belowground carbon; SOC: soil organic carbon; TC: Total carbon. GRSP: Glomalin Related Soil Proteins; AMF: Number of Arbuscular Mycorrhizal fungal spores.

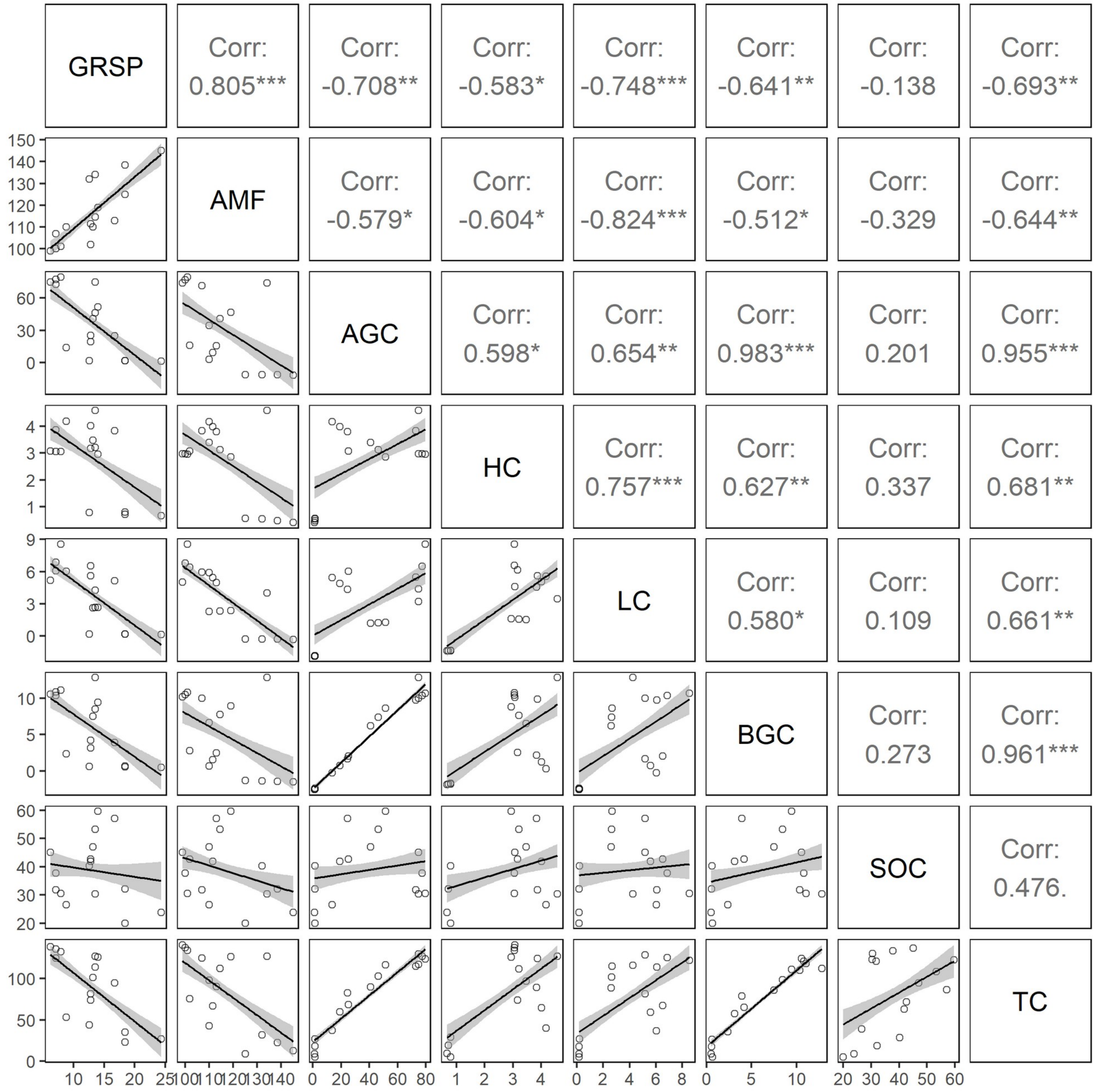

Supplementary Figure 2. Correlogram showing correlation coefficients between Glomalin Related Soil Proteins (GRSP) and number of Arbuscular Mycorrhizal Fungal (AMF) spores per 25 g of soil with carbon components. AGC: aboveground carbon; HC: herbaceous carbon; LC: leaf litter carbon; BGC: belowground carbon; SOC: soil organic carbon; TC: Total carbon;

Supplementary Table 1. Allometric equations used to estimate aboveground and belowground biomass.

Group of species	Barometric model	r^2	DBH range (cm)	References
Tropical rainforest	AGB = exp (−1.7689 + 2.377 × Ln (DBH))	0.96	$2 \leq DBH < 5$	Nascimento and Laurance (2002)
Tropical rainforest	$AGB = 0.0673 \times (\rho \times DBH^2 \times H)^{0.976}$		$5 \leq DBH$	Chave *et al*. (2014)
Inga sp.	$AGB = 0.01513 \times DBH^{3.0054}$	0.83	$10 \leq DBH \leq 29$	Castellanos *et al*. (2010)
Coffea arabica	$Ln(AGB) = -2.39287 + 0.95285 \times Ln(d_{15}) + 1.2693 \times Ln(H)$	0.63	$0 \leq d_{15} \leq 9$	Suarez (2004)
Roots	Y = exp (−1.0587 + 0.8836 × Ln (AGB))	0.84	-	Cairns *et al*. (1997)

AGB: aboveground biomass; Y: root biomass; DBH: diameter at breast height (1.3 meters); d15: diameter at 15 cm above ground; H: height (m); and ρ: wood density.
